# Assessment of Serum 3‐Epi‐25‐Hydroxyvitamin D_3_ , 25‐Hydroxyvitamin D_3_ and 25‐Hydroxyvitamin D_2_ in the Korean Population With UPLC–MS/MS

**DOI:** 10.1002/jcla.70098

**Published:** 2025-09-12

**Authors:** Sung‐Eun Cho, Jungsun Han, Gayoung Chun, Rihwa Choi, Sang Gon Lee, Hyung‐Doo Park

**Affiliations:** ^1^ Endocrine Substance Analysis Center (ESAC) GC Labs Gyeonggi‐do Korea; ^2^ GC Labs Gyeonggi‐do Korea; ^3^ Infectious Disease Research Center GC Labs Gyeonggi‐do Korea; ^4^ Department of Laboratory Medicine GC Labs Gyeonggi‐do Korea; ^5^ Department of Laboratory Medicine and Genetics Samsung Medical Center, Sungkyunkwan University School of Medicine Seoul Korea

**Keywords:** 3‐epi‐25‐OH‐D_3_, KNHANES, tandem mass spectrometry

## Abstract

**Background:**

The liquid chromatography–tandem mass spectrometry method (LC–MS/MS) identified an epimeric form of 25‐hydroxyvitamin D_3_ (3‐epi‐25‐OH‐D_3_) concentrations. For the first time, the 3‐epi‐25‐OH‐D_3_ was included in the Korea National Health and Nutrition Examination Survey (KNHANES) IX (from 2022 to 2024). In this study, we evaluated the results of 3‐epi‐25‐OH‐D_3_ in KNHANES IX to determine its clinical status in Korea.

**Methods:**

We measured 3‐epi‐OH‐D_3_, 25‐OH‐D_3_, and 25‐OH‐D_2_ concentrations using an Ultra Performance (UP) LC–MS/MS, and assessed the differences in concentration ranges based on gender and age among participants in this national project.

**Results:**

The concentration range of 3‐epi‐25‐OH‐D_3_ among 11,744 Korean participants (5193 male and 6551 female, aged 10–80 with a median age of 53) was 0.50 (lower limit of quantification) to 15.66 ng/mL, with a mean ± SD of 1.15 ± 0.85 ng/mL. The 3‐epi‐25‐OH‐D_3_ concentration was not significantly different between genders. The concentrations of 25‐OH‐D metabolites were different across age groups. There was a highly significant correlation between 3‐epi‐25‐OH‐D_3_ and 25‐OH‐D_3_ concentrations (*r* = 0.77, *R* ^2^ = 0.5911, slope = 0.0579, *p* < 0.0001). The minimum and maximum of % 3‐epi‐25‐OH‐D_3_ were 1.33% and 23.94%, respectively. The % 3‐epi‐25‐OH‐D_3_ was highest in the highest quartile (Q4) of the total 25‐OH‐D_3_ concentration (*p* < 0.0001).

**Conclusions:**

This is the first large‐scale study of 3‐epi‐25‐OH‐D_3_ concentrations in the Korean population. Accurate measurement of 3‐epi‐25‐OH‐D_3_ is necessary for the reliable results of vitamin D metabolites in KNHANES.

## 1 Introduction

Vitamin D is an essential nutrient important in calcium and phosphorus metabolism, maintaining bone health, and regulating the immune system [1, 2]. Vitamin D deficiency has been reported to be associated with osteoporosis, osteomalacia, immune disease, cardiovascular disease, and some types of cancers, and various studies have been conducted, especially among the elderly, adolescents, obese people, and people with certain chronic diseases [3, 4, 5, 6, 7, 8, 9, 10].

Depending on the intake source, vitamin D exists as either vitamin D_3_ (cholecalciferol) or vitamin D_2_ (ergocalciferol), and most vitamin D in the prohormone state is converted to 25‐hydroxyvitamin D (25‐OH‐D), the main form in systemic circulation [11]. 25‐OH‐D is the precursor to active 1,25‐dihydroxyvitamin D (1,25‐OH‐D) and has a longer half‐life. There are two types of 25‐OH‐D, which are 25‐OH‐D_3_ and 25‐OH‐D_2_. 25‐OH‐D_3_ is more potent and is normally present in higher body concentrations than 25‐OH‐D_2_ [12].

Immunoassays are common methods for measuring vitamin D in clinical laboratories. However, immunoassays rely on the interaction of 25‐OH‐D metabolites with antibodies that may lack specificity, and this methodology faces challenges with interfering compounds. So, the reference measurement procedure (RMP) for 25‐OH‐D is the isotope‐dilution liquid chromatography–tandem mass spectrometry (ID‐LC–MS/MS) method with more specificity and accuracy [13, 14, 15].

In 2006, Singh et al. developed an LC–MS/MS method to identify an epimeric form of 25‐OH‐D_3_. They reported that 3‐epi‐25‐OH‐D_3_ is found in 23% of infants and contributes 9%–61% of the total 25‐OH‐D metabolites [16]. Numerous studies have reported elevated levels of 3‐epi‐25‐OH‐D in various conditions and diseases. However, the potential association between 3‐epi‐OH‐D and diseases remains largely unknown [17].

C3‐epimers are a vitamin D metabolite produced by the epimerization pathway. In the epimerization pathway, the intermediate metabolites of major vitamin D precursors are converted to epimers by 3‐epimerase. These epimers undergo the same hydroxylation and oxidation events by the same enzymes as the standard metabolic pathway, producing the epimers. Although the potential association between 3‐epi‐OH‐D and diseases remains largely unknown, the C3‐epimers have been shown to possess some calcemic and non‐calcemic regulatory effects compared to their non‐epimeric form in calcium, phosphorus, and PTH homeostasis [18].

Since 2007, the determination of 3‐epi‐25‐OH‐D_3_ has been nationally surveyed in the USA's National Health and Nutrition Examination Survey (NHANES) using the LC–MS/MS method. The analysis of serum 25‐OH‐D for all of the NHANES surveys was conducted in Atlanta, GA, at the National Center for Environmental Health (NCEH) within the Centers for Disease Control and Prevention (CDC). Although the clinical significance of the epimeric form has not yet been established, failure to separate it when measuring total vitamin D can lead to falsely elevated results. For this reason, the U.S. National Health and Nutrition Examination Survey (NHANES) adopted an LC–MS/MS method capable of separating epimers, enabling the calculation of an accurate total vitamin D concentration by summing only vitamin D_2_ and D_3_, excluding the epimeric form [19].

In Korea, the Korea National Health and Nutrition Examination Survey (KNHANES) has been conducted since 1998, and vitamin D was included in this survey program from 2007 to 2014. The measurement method of vitamin D was radioimmunoassay (RIA) [20].

Then, the analysis of vitamin D was started again from the KNHANES IX (from 2022 to 2024), in which the 3‐epi‐25‐OH‐D_3_ was included for the first time in Korea. For the analysis of 3‐epi‐25‐OH‐D_3_, the measurement method had to be changed from RIA to LC–MS/MS.

GC Labs Endocrine Substance Analysis Center (ESAC) measured the serum‐25‐OH‐D metabolites, including 3‐epi‐25‐OH‐D_3_, with an Ultra Performance (UP) LC–MS/MS method for this national KNHANES project and is now evaluating the results to determine its clinical status in Korea.

## 2 Methods

This evaluation study was conducted from October 2024 to February 2025, following approval from the Institutional Review Board (IRB) of GC Labs (IRB No. GCL‐2024‐1079‐01).

### 2.1 Data Collection From the KNHANES IX 2022–2023

KNHANES is a nationwide survey conducted under Article 16 of the National Health Promotion Act to assess the Korean population's health status, health behaviors, and dietary and nutritional intake.

Among the results of the KNHANES IX project conducted from 2022 to 2024, we evaluated the data from 2022 to 2023, during which we measured the concentrations of serum 25‐OH‐D metabolites from January 2022 to December 2023.

The KNHANES publicly releases data that does not contain personally identifiable information through its website. Although we measured and produced the above raw data, we did not use it. Instead, we cited and used the KCDC National Health Statistics 2022 and 2023 data, released on its website before January 2025. A detailed description of the methodology of KNHANES, including the sampling procedure and sample recruitment, was reported through its website [21].

In summary, the KNHANES dataset includes only quality‐assured specimen results, as verified through the “Quality Control of the Clinical Laboratory for the KNHANES (2022‐2023, 9th)” study [21]. In the present study, we utilized the entire dataset. Pre‐analytical processing was performed immediately inside the mobile examination vehicle after blood collection. A volume of 8 mL of blood was drawn into an 8.5 mL serum separator tube (Becton, Dickinson and Company [BD] SST no. 367953, Franklin Lakes, USA), gently inverted five times, and left to stand at room temperature for 30 min. The sample was then centrifuged at 1300 x g (approximately 3000 rpm) for 10 min and stored at 2°C–8°C. It was analysed on the same day, within 24 h, at GC Labs for all analytes except 3‐epi‐25‐OH‐D_3_, 25‐OH‐D_3_, and 25‐OH‐D_2_. Samples for 3‐epi‐25‐OH‐D_3_, 25‐OH‐D_3_, and 25‐OH‐D_2_ were stored at −70°C until analysis at GC Labs ESAC within 1 month.

A total of 13,194 participants were selected nationwide for KNHANES in 2022 and 2023, and serum vitamin D levels were measured in 11,744 of them [21]. The total number of available vitamin D results was 11,744, comprising 5193 males and 6551 females, aged 10–80, and a median age of 53.

### 2.2 Analysis Items

The analyses items were 3‐epi‐25‐OH‐D_3_, 25‐OH‐D_3_, 25‐OH‐D_2_, total cholesterol, high‐density lipoprotein (HDL) cholesterol, triglyceride, low‐density lipoprotein (LDL) cholesterol, blood urea nitrogen (BUN), creatinine, glucose, hemoglobin A1c (HbA1c), alkaline phosphatase (ALP), uric acid, aspartate aminotransferase (AST), alanine aminotransferase (ALT), high‐sensitivity C‐reactive protein (hs‐CRP), hemoglobin (Hb), hematocrit (Hct), white blood cell count (WBC), red blood cell count (RBC), and platelet count (Plt), which were all measured in GC Labs.

### 2.3 Reagents and Materials for Measuring Vitamin Ds

The MSMS Vitamin D Kit (PerkinElmer, Turku, Finland) consisted of calibrators with six levels, quality control (QC) materials with three levels, internal standards (ISs), a derivatization box, and a toolbox. The toolbox contained microplates and plate covers used during sample preparation. The derivatization box included the derivatization reagent (PTAD; 4‐phenyl‐1,2,4‐triazoline‐3,5‐dione), a quenching solution, and an HPLC solvent additive.

Additional materials not provided with the kit included HPLC‐grade water, acetonitrile, and methanol (all from Fisher Scientific, Pittsburgh, USA); formic acid (Wako, Osaka, Japan); and Standard Reference Material (SRM) 972a, which contains vitamin D metabolites in frozen human serum (National Institute of Standards and Technology (NIST), Gaithersburg, USA).

### 2.4 Calibrators and QC Materials for Measuring Vitamin Ds

Two additional low‐level calibrators were prepared by diluting the calibrator level 6 at 1:100 and 1:50, respectively. As a result, the final concentrations of calibrators for 25‐OH‐D_2_ were 1.37, 2.75, 4.17, 8.38, 17.17, 34.79, 68.51, and 137.43 ng/mL. For both 25‐OH‐D_3_ and 3‐epi‐25‐OH‐D_3_, the calibrator concentrations were 1.31, 2.61, 3.81, 7.73, 16.14, 32.97, 65.70, and 130.60 ng/mL. The manufacturer did not disclose the concentration of the ISs.

Three QC samples were used to assess concentrations of low, medium, and high levels. The QC concentrations for 25‐OH‐D_2_ were 8.34, 34.58, and 69.33 ng/mL, and for 25‐OH‐D_3_ were 7.77, 32.90, and 66.12 ng/mL, respectively.

In addition, SRM 972a (NIST, Gaithersburg, USA) was analyzed in each batch as an internal QC material, too.

### 2.5 Sample Pretreatment for Measuring Vitamin Ds

The sample pretreatment steps included protein precipitation, solvent extraction, followed by derivatization.

Frozen calibrators, QC materials, NIST SRM 972a, and patient samples were thawed at room temperature and thoroughly mixed. A 100 μL aliquot of each sample was treated with 200 μL of daily precipitation solution (DPS) containing ISs to induce protein precipitation, then centrifuged. After centrifugation, 150 μL of the supernatant was transferred and evaporated to dryness. The dried residue was derivatized with PTAD, followed by quenching. The final solution was filtered through a 0.2 μm regenerated cellulose (RC) membrane filter (Minisart RC4) (Sartorius AG, Göttingen, Germany). 2 μL of an aliquot of the final extract was used for analysis.

### 2.6 UPLC–MS/MS Setting Conditions and Assay Performance Data

The samples were introduced into a Nexera‐X2‐LC‐30ad UPLC (Shimadzu, Tokyo, Japan) system equipped with a Kinetex XB C18 column (2.1 × 150 mm, 2.6 μm; Phenomenex, Torrance, USA) on a Triple Quad 4500MD (Sciex, Framingham, USA) MS/MS system. This method had a longer runtime of 16 min (compared to 4.5 min without measuring 3‐epi‐25‐OH‐D). The chromatographic conditions of UPLC–MS/MS are provided in Table S1. The MRM transitions and other MS/MS setting conditions are provided in Table S2. The representative chromatograms of serum 25‐OH‐D_2_, 25‐OH‐D_3_, and 3‐epi‐25‐OH‐D_3_ are shown in Figure S1. The intra‐ and inter‐run precision and accuracy of this method are indicated in Table S3. The actual QC chart summaries using SRMs as control materials are shown in Table S4.

### 2.7 Data Reporting Format

All 25‐OH‐D measurements were reported in conventional units (ng/mL), with 1 ng/mL corresponding to a SI unit of 2.423 nmol/L for 25‐OH‐D_2_ and 2.496 nmol/L for 25‐OH‐D_3_, respectively [22].

We used the lower limit of quantification (LLOQ) value as the imputed value for 25‐OH‐D_2_, 25‐OH‐D_3_, and 3‐epi‐25‐OH‐D_3_ when the concentrations were less than the LLOQ, which were 0.30, 0.29, and 0.50 ng/mL, respectively.

### 2.8 Measurement Methods for Other Markers

The measurement method for total cholesterol, HDL cholesterol, LDL cholesterol, BUN, creatinine, glucose, ALP, uric acid, AST, ALT, and hs‐CRP was performed using Cobas 8000 (Roche Diagnostics, Indianapolis, USA) with dedicated reagents for each item. The measurement method for HbA1c was performed using Tosoh G11 (Tosoh Corporation, Tokyo, Japan) systems, equipped with dedicated reagents. HbA1c was measured using the Tosoh G11 system (Tosoh Corporation, Tokyo, Japan) with dedicated reagents. Hb, Hct, WBC, RBC, and Plt were measured using the Sysmex XN‐1000 analyzer (Sysmex Corporation, Kobe, Japan).

### 2.9 Statistical Analysis

The KNHANES provides guidelines for proper statistical analysis methods (such as applying various weights) in a separate data analysis manual (Raw Data Usage Guidelines) posted on the same website. Therefore, statistical analysis was conducted following these guidelines [21].

The statistical analysis methods are as follows: The 3‐epi‐25‐OH‐D_3_, 25‐OH‐D_3_, and 25‐OH‐D_2_ were evaluated based on the participants' gender and age group characteristics. The differences in concentration values between genders were analyzed using a *T*‐test or Wilcoxon rank‐sum test, and the differences between age groups were tested using the Kruskal–Wallis test or one‐way ANOVA followed by an *F*‐test. Additionally, Spearman and Pearson correlation analyses were conducted to assess the associations between 3‐epi‐25‐OH‐D_3_ or % 3‐epi‐25‐OH‐D_3_ and other markers, adjusting for age and gender. We did not apply any normalization or transformation to address skewed distributions.

The statistical analysis was performed using SAS (version 9.4, SAS Institute Inc., Cary, NC, USA), and all tests with a *p* value of < 0.05 were considered statistically significant.

### 2.10 Thresholds of 25‐OH‐D Deficiency, Insufficiency, and Optimal Concentrations

We defined the thresholds for 25‐OH‐D deficiency, insufficiency, suboptimal, optimal, and toxic concentrations as < 10, < 20, < 30, ≥ 30, ≥ 100 ng/mL, respectively, following the previous studies in Korea [23, 24].

## 3 Results

### 3.1 Overall Distribution of Serum Vitamin D Metabolites in KNHANES IX 2022–2023

The concentration ranges of 25‐OH‐D_2_, 3‐epi‐25‐OH‐D_3_, 25‐OH‐D_3_, and total 25‐OH‐D (25‐OH‐D_2_ + 3‐epi‐25‐OH‐D_3 +_ 25‐OH‐D_3_) were 0.30 (LLOQ) to 22.13, 0.50 (LLOQ) to 15.66, 2.82–128.46, 3.62–140.44 ng/mL, respectively. We found quantifiable 25‐OH‐D_3_ in 100% of the population, but in cases of 25‐OH‐D_2_ and 3‐epi‐25‐OH‐D_3_, 46.7% and 16.7% of the population had lower or the same concentrations of LLOQ, respectively (Table 1).

**TABLE 1:** Overall distribution and concentrations of 25‐OH‐D metabolites, including 3‐epi‐25‐OH‐D_3_, according to gender and age in KNHANES IX 2022–2023.

	Vitamin D measured participants (*n*)	25‐OH‐D_2_		3‐epi‐25‐OH‐D_3_		25‐OH‐D_3_		25‐OH‐D_2_ + 25‐OH‐D_3_		Total vitamin D (25‐OH‐D_2_ + 3‐epi‐25‐OH‐D_3_ + 25‐OH‐D_3_)	
		Mean ± SD (ng/mL)	*p*	Mean ± SD (ng/mL)	*p*	Mean ± SD (ng/mL)	*p*	Mean ± SD (ng/mL)	*p*	Mean ± SD (ng/mL)	*p*
*Sex*											
M	5193	0.41 ± 0.59	0.1047^a^	1.13 ± 0.78	0.1933^a^	22.50 ± 10.12	< 0.0001^a^	22.92 ± 10.12	< 0.0001^a^	24.05 ± 10.72	< 0.0001^a^
F	6551	0.45 ± 0.64		1.17 ± 0.89		24.76 ± 11.96		25.21 ± 11.94		26.38 ± 12.65	
Age											
< 20	964	0.41 ± 0.39	0.0007^b^	0.91 ± 0.56	< 0.0001^b^	18.42 ± 7.65	< 0.0001^b^	18.83 ± 7.63	< 0.0001^b^	19.74 ± 8.09	< 0.0001^b^
20–29	1112	0.39 ± 0.39		0.84 ± 0.62		17.40 ± 8.69		17.80 ± 8.68		18.63 ± 9.19	
30–39	1312	0.43 ± 0.60		0.98 ± 0.66		21.07 ± 9.58		21.50 ± 9.58		22.48 ± 10.11	
40–49	1805	0.41 ± 0.41		0.99 ± 0.60		21.85 ± 9.58		22.26 ± 9.57		23.26 ± 10.04	
50–59	1973	0.41 ± 0.43		1.19 ± 0.83		24.69 ± 11.28		25.10 ± 11.28		26.29 ± 11.92	
60–69	2386	0.47 ± 0.86		1.42 ± 1.06		27.77 ± 11.78		28.24 ± 11.72		29.66 ± 12.51	
≥ 70	2192	0.47 ± 0.76		1.34 ± 0.94		27.30 ± 12.07		27.78 ± 12.06		29.12 ± 12.78	
Total	11,744	0.44 ± 0.62		1.15 ± 0.85		23.76 ± 11.24		24.19 ± 11.23		25.35 ± 11.89	

Abbreviation: SD, standard deviation.

^a^ This *p*‐value is from the Wilcoxon rank‐sum test.

^b^ This *p*‐value is from the Kruskal–Wallis test.

### 3.2 Concentrations of 25‐OH‐D Metabolites According to Gender

The concentrations of 25‐OH‐D metabolites according to gender are as follows. The concentrations of 25‐OH‐D_2_, 3‐epi‐25‐OH‐D_3_, 25‐OH‐D_3_, and total 25‐OH‐D were 0.41 ± 0.59, 1.13 ± 0.78, 22.50 ± 10.12, and 24.05 ± 10.72 for males, and 0.45 ± 0.64, 1.17 ± 0.89, 24.76 ± 11.96, and 26.38 ± 12.65 ng/mL for females, respectively. The concentrations of 25‐OH‐D_3_ and total 25‐OH‐D were significantly higher in females than in males (*p* < 0.0001), but the concentrations of 25‐OH‐D_2_ and 3‐epi‐25‐OH‐D_3_ were not significantly different between males and females (Table 1).

### 3.3 Concentrations of 25‐OH‐D Metabolites According to Age

The concentrations of 25‐OH‐D metabolites according to age are as follows. The blood concentrations of 25‐OH‐D metabolites differed across age groups (*p* < 0.0001). The 25‐OH‐D_2_, 3‐epi‐25‐OH‐D_3_, 25‐OH‐D_3_, and total 25‐OH‐D were highest in the 60s, followed by higher concentrations in the age group of 70s and above. In those under 60, the concentrations tended to increase according to the age increase (Table 1).

### 3.4 The Correlation Between 3‐Epi‐25‐OH‐D_3_ and 25‐OH‐D_3_

**FIGURE 1:**
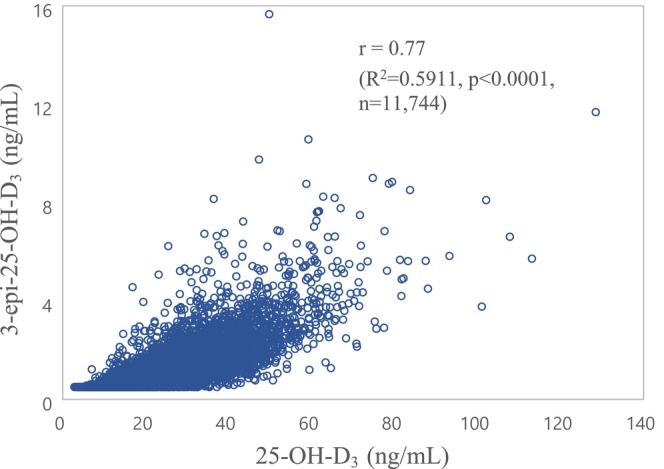
Scatter plot of serum 3‐epi‐25‐OH‐D_3_ and 25‐OH‐D_3_ concentrations. There is a significant correlation between these two metabolites.

The correlation between serum 3‐epi‐25‐OH‐D_3_ and 25‐OH‐D_3_ concentration is shown in Figure 1.

There was a highly significant correlation between these two metabolites (Pearson's correlation coefficient, *r* = 0.77; *p* < 0.0001), and the linear relation was as follows: *R* ^2^ = 0.5911, *p* < 0.0001, *n* = 11,744.

(serum 3‐epi‐25‐OH‐D_3_) = 0.0579 × (serum 25‐OH‐D_3_) – 0.17285.

(Figure 1).

### 3.5 The Serum 3‐epi‐25‐OH‐D_3_ Concentrations and % 3‐epi‐OH‐D_3_ According to Quartile of Serum Total 25‐OH‐D_3_

The mean ± SD of 3‐epi‐25‐OH‐D_3_ concentrations and % 3‐epi‐25‐OH‐D_3_ by quartile of serum total 25‐OH‐D_3_ concentration are shown in Table 2. The total 25‐OH‐D_3_ concentration was the sum of the 3‐epi‐25‐OH‐D_3_ and 25‐OH‐D_3_ concentrations. % 3‐epi‐OH‐D_3_ was calculated as below:

**TABLE 2:** Serum 3‐epi‐25‐OH‐D_3_ concentration and % 3‐epi‐25‐OH‐D_3_ by quartile of serum total 25‐OH‐D_3_ concentration.

	Quartiles of total 25‐OH‐D_3_ concentration* (ng/mL)				*p* ^†^
	Minimum to Q1	Q1–Q2	Q2–Q3	Q3 to maximum	
	3.32–16.02	16.02–23.36	23.36–31.49	31.49–140.14	
Participants (*n*) (total *n* = 11,744)	2933	2939	2932	2940	
3‐epi‐25‐OH‐D_3_ (ng/mL)^‡^	0.56 ± 0.13^a^	0.82 ± 0.28^b^	1.18 ± 0.41^c^	2.06 ± 1.15^d^	< 0.0001
% 3‐epi‐25‐OH‐D_3_ (%)^∬^	4.91 ± 1.37^a^	4.16 ± 1.32^b^	4.32 ± 1.45^c^	4.92 ± 1.95^d^	< 0.0001

*Note:* (a, b, c, d) are significantly different from each other (*p* < 0.05).

Abbreviation: SD, standard deviation.

* The total 25‐OH‐D_3_ concentration = 3‐epi‐25‐OH‐D_3_ + 25‐OH‐D_3_.

^†^ This *p* value is from the Kruskal–Wallis test. The post hoc test uses the Dwass, Steel, Critchlow‐Fligner (DSCF) multiple comparison procedure.

^‡^ Values are means ± SDs.

^∬^ % 3‐epi‐25‐OH‐D_3_ = (3‐epi‐25‐OH‐D_3_/total 25‐OH‐D_3_) × 100.

(% 3‐epi‐25‐OH‐D_3_) = (3‐epi‐25‐OH‐D_3_/Total 25‐OH‐D_3_) × 100

The minimum, median, and maximum concentrations of total 25‐OH‐D_3_ were 3.32, 23.36, and 140.14 ng/mL, respectively. The minimum and maximum 3‐epi‐25‐OH‐D_3_ concentrations were 0.50 and 15.66 ng/mL, respectively. The minimum and maximum percentages of 3‐epi‐25‐OH‐D_3_ were 1.33% and 23.94%, respectively. The mean 3‐epi‐25‐OH‐D_3_ concentration increased linearly by quartile of serum total 25‐OH‐D_3_ concentration except for the Q1 quartile, and the % 3‐epi‐25‐OH‐D_3_ was highest in the Q4 quartile of total 25‐OH‐D_3_ concentration (*p* < 0.0001) (Table 2).

### 3.6 Impact of Epimer Inclusion in Serum Total 25‐OH‐D on the Prevalence of Vitamin D Deficiency/Inadequacy

The percentage of participants with serum total 25‐OH‐D below variously proposed thresholds of serum 25‐OH‐D and the impact of including the serum 3‐epi‐25‐OH‐D_3_ concentration on it are shown in Table 3. Including 3‐epi‐25‐OH‐D_3_ concentration in the estimate of serum total 25‐OH‐D reduced the prevalence of serum 25‐OH‐D deficiency, insufficiency, and suboptimal levels by 1.1, 2.8, and 4.1 percentage points, respectively. The prevalence of optimal levels increased from 26.3% to 30.3%, and the number of participants with toxic levels remained unchanged when the 3‐epi‐25‐OH‐D_3_ concentration was included in the total estimate (Table 3).

**TABLE 3:** Population with serum total 25‐OH‐D below various thresholds, with and without the inclusion of serum 3‐epi‐OH‐D_3_.

Threshold (ng/mL)	Participants (*n*)	Total 25‐OH‐D					
		Without 3‐epimer (=25‐OH‐D_2_ + 25‐OH‐D_3_)			With 3‐epimer (=25‐OH‐D_2_ + 25‐OH‐D_3_ + 3‐epi‐25‐OH‐D_3_)		
		*n*	%	95% CI	*n*	%	95% CI
< 10	11,744	761	6.5	6.0–6.9	635	5.4	5.0–5.8
< 20	11,744	4703	40.1	39.2–40.9	4384	37.3	36.5–38.2
< 30	11,744	8651	73.7	72.9–74.5	8176	69.6	68.8–70.5
30–100	11,744	3088	26.3	25.5–27.1	3563	30.3	29.5–31.2
≥ 100	11,744	5	0.0	0.0–0.1	5	0.0	0.0–0.1

Abbreviation: CI, confidence interval.

### 3.7 Correlation Between 3‐Epimers and Other Blood Markers of KNHANES IX 2022–2023

Table 4 presents the correlations between the 3‐epimers (3‐epi‐25‐OH‐D_3_ or % 3‐epi‐25‐OH‐D_3_) and other blood markers of KNHANES IX 2022–2023 data, after adjustment for age and gender. All markers except Hct, glucose, and TG showed significant correlations with 3‐epi‐25‐OH‐D_3_ concentration. Additionally, all markers, except hs‐CRP, had significant correlations with % 3‐epi‐25‐OH‐D_3_. Among them, Plt, BUN, creatinine, total cholesterol, and LDL cholesterol showed a negative correlation with % 3‐epi‐25‐OH‐D_3_ (Table 4).

**TABLE 4:** Correlation between 3‐epimers and other blood markers of KNHANES IX 2022–2023.

Blood markers	Participants (*n*)	3‐epi‐25‐OH‐D_3_		% 3‐epi‐25‐OH‐D_3_	
		Spearman's correlation coefficient^a^	*p*	Spearman's correlation coefficient^a^	*p*
WBC	11,732	−0.02384	0.0098	0.04666	< 0.0001
RBC	11,732	−0.02137	0.0207	0.02060	0.0256
PLT	11,681	−0.04489	< 0.0001	−0.03318	0.0003
Uric acid	11,743	0.03056	0.0009	0.02827	0.0022
Hs‐CRP	11,744	−0.05147	< 0.0001	−0.01606	0.0818
Hb	11,732	0.03967	< 0.0001	0.05002	< 0.0001
HCT	11,732	0.01714	0.0635	0.03428	0.0002
BUN	11,744	0.06028	< 0.0001	−0.07113	< 0.0001
Creatinine	11,744	−0.02344	0.0111	−0.07044	< 0.0001
Glucose	11,744	−0.00007	0.9936	0.06921	< 0.0001
HbA1C	11,710	0.04225	< 0.0001	0.08902	< 0.0001
Total cholesterol	11,744	−0.06773	< 0.0001	−0.17844	< 0.0001
HDL cholesterol	11,744	0.10361	< 0.0001	0.01815	0.0492
LDL cholesterol	11,744	−0.10701	< 0.0001	−0.22530	< 0.0001
TG	11,744	−0.00582	0.5284	0.08088	< 0.0001
AST	11,743	0.17744	< 0.0001	0.15718	< 0.0001
ALT	11,739	0.12076	< 0.0001	0.11277	< 0.0001

*Note:* *p* < 0.05 indicates significance.

^a^ This analysis was adjusted for age and gender with a partial correlation coefficient.

## 4 Discussion

We evaluated the results of 3‐epi‐25‐OH‐D_3_ in KNHANES 2022–2023 to understand its clinical status in Korea. We set up the UPLC–MS/MS method for measuring 3‐epi‐OH‐D_3_ and were able to provide the KNHANES data using this method.

This is the first large‐scale study on 3‐epi‐25‐OH‐D_3_ serum concentrations across all age groups over 10 years old in the Korean population. Previously, there was only a study on 3‐epi‐25‐OH‐D_3_ concentrations in Korean infants, pediatric, and adolescent populations [25], as well as another on its role as an endogenous interferent in various immunoassay and mass spectrometry methods in Korea [26].

Our method differs from conventional LC–MS/MS methods for measuring vitamin D. Other traditional LC–MS/MS methods can't separate 3‐epi‐25‐OH‐D_3_ [26]. To separate 3‐epi‐25‐OH‐D_3_, a more complex and complicated method must be set up in the clinical laboratory. The LC–MS/MS method for separating 3‐epi‐25‐OH‐D_3_ requires a longer separation time with a more extended LC column, an extensive sample preparation method, or a high‐resolution mass spectrometer [25].

The levels of 3‐epi‐25‐OH‐D_3_ production could be related to gender, age, and living areas [27, 28]. However, results on these factors vary among researchers. Chailurkit et al. reported higher concentrations of the vitamin D epimer in males than in females [28]. Another study found that pregnant women and newborns had higher levels of C3‐epimers [29]. In infants, particularly in the first 3 months, 3‐epi‐25‐OH‐D_3_ concentrations were significantly higher, representing up to 60% of total 25‐OH‐D [16]. It was concluded that the epimer levels decrease as infants grow older, remaining approximately constant throughout adulthood [18].

Our results on the epimer concentrations in the Korean population were comparable with previous studies conducted in other countries. In our study, the concentration range of 3‐epi‐25‐OH‐D_3_ was from 0.50 (LLOQ) to 15.66 ng/mL, with a mean ± SD of 1.15 ± 0.85 ng/mL. We found quantifiable 25‐OH‐D_2_ and 3‐epi‐25‐OH‐D_3_ in 53.3% and 83.3% of the population, respectively.

In a study of a nationally representative sample of adults in Ireland, the mean concentration of 3‐epi‐25‐OH‐D3 was 2.5 nmol/L (1.0 ng/mL) [30], which is similar to our findings.

In the previous NHANES (2007–2010) study in the USA, 25‐OH‐D_3_, 25‐OH‐D_2_, and 3‐epi‐25‐OH‐D_3_ were measured using the LC–MS/MS method in 15,652 participants who provided serum samples for 25‐OH‐D testing over 4 years. The 3‐epi‐25‐OH‐D_3_ concentrations ranged from <limit of detection (LOD) (1.64 nmol/L [0.66 ng/mL]) to 37.8 nmol/L (15.12 ng/mL), which is similar to the range observed in our study. They detected 25‐OH‐D_2_ and 3‐epi‐25‐OH‐D_3_ in 19% and 86%, respectively [31]. These differences in the detection rates of 25‐OH‐D_2_ compared with our study are likely explained by variations in the LLOQ or LOD between the two studies.

In our study, we used the LLOQ as the input value for 25‐OH‐D_2_, 25‐OH‐D_3_, and 3‐epi‐25‐OH‐D_3_ when the measured concentrations were below their respective LLOQs, which were 0.30, 0.29, and 0.50 ng/mL, respectively. No samples had 25‐OH‐D_3_ concentrations below the LLOQ. For 25‐OH‐D_2_ and 3‐epi‐25‐OH‐D_3_, inputting values below the LLOQ as the LLOQ may lead to an overestimation of the mean concentrations and detection rates, particularly for 3‐epi‐25‐OH‐D_3_. However, our LLOQ for 3‐epi‐25‐OH‐D_3_ (0.50 ng/mL) was even lower than the LOD used in the NHANES study conducted in the United States (0.66 ng/mL). Furthermore, the detection rate of 3‐epi‐25‐OH‐D_3_ in our study was similar to that reported in NHANES (83.3% vs. 86%). In a recent update to our method, we further lowered the LLOQ for 3‐epi‐25‐OH‐D_3_ from 0.50 to 0.30 ng/mL. Therefore, we expect the detection rate to increase even more, suggesting that the potential overestimation of concentration due to imputation is likely minimal.

We also compared our results for epimer concentrations by gender with previous studies [17, 30]. In our study, the concentrations of 25‐OH‐D_3_ and total 25‐OH‐D were significantly higher in females than in males, while the concentrations of 25‐OH‐D_2_ and 3‐epi‐25‐OH‐D_3_ showed no significant difference between genders. The total 25‐OH‐D concentration is primarily influenced by 25‐OH‐D_3_, which has the highest concentration, while 25‐OH‐D_2_ and 3‐epi‐25‐OH‐D_3_, due to their low concentrations, do not appear to affect the total 25‐OH‐D levels. This study is the first to demonstrate that epimers show no gender differences in the Korean population.

In the other study, the gender difference was observed not in the percentage of 3‐epi‐25‐OH‐D_3_ but in the concentrations of 3‐epi‐25‐OH‐D_3_ [17]. In a survey of adults in Ireland, no gender difference was found in the 3‐epi‐25‐OH‐D_3_ concentrations [30].

In our study, the concentrations of the 3‐epi‐25‐OH‐D_3_ below 20 were slightly higher than those of the 20s. The concentrations of 25‐OH‐D_2_, 3‐epi‐25‐OH‐D_3_, 25‐OH‐D_3_, and a total of 25‐OH‐D in the age group of 10–20 years old in our study were similar to the previous study in Korea within the same age group of 10–20 years old measured by LC–MS/MS with the epimer separation method [25] (Table 5). In the study of adults in Ireland, serum 3‐epi‐25‐OH‐D_3_ concentrations were lowest in the below‐50s age group and highest in the 65sand above age group [30], similar to our results.

**TABLE 5:** Comparison of concentrations of 25‐OH‐D metabolites, including 3‐epi‐25‐OH‐D_3_, with the previous study [25].

	Age of participants (years)	Vitamin D measured participants (*n*)	25‐OH‐D_2_	3‐epi‐25‐OH‐D_3_	25‐OH‐D_3_	25‐OH‐D_2_ +25‐OH‐D_3_	Total vitamin D^a^
			Mean ± SD (ng/mL)	Mean ± SD (ng/mL)	Mean ± SD (ng/mL)	Mean ± SD (ng/mL)	Mean ± SD (ng/mL)
Previous study [25]	10–20	207	0.30 ± 0.59^b^	0.62 ± 0.35^b^	17.38 ± 6.92^b^	17.67 ± 7.01^b^	18.29 ± 7.22^b^
This study	10–20	964	0.41 ± 0.39	0.91 ± 0.56	18.42 ± 7.65	18.83 ± 7.63	19.74 ± 8.09

Abbreviation: SD, standard deviation.

^a^ Total vitamin D means the sum of 25‐OH‐D_2_, 3‐epi‐25‐OH‐D_3_, and 25‐OH‐D_3_ concentrations.

^b^ This value is converted from nmol/L to ng/mL.

There was a strong relation between serum 3‐epi‐25‐OH‐D_3_ and 25‐OH‐D_3_ concentrations. We obtained a correlation coefficient (r) of 0.77 (*p* < 0.0001), which is similar to the previous study, reporting a strong correlation between the two markers with a correlation coefficient (*r*) of 0.77 (*p* < 0.0001) [31].

Furthermore, the mean 3‐epi‐25‐OH‐D_3_ concentration increased by a quartile of serum total 25‐OH‐D_3_ concentration, similar to that of the previous study [30]. The % 3‐epi‐25‐OH‐D_3_ result in our study was highest in the highest quartile (Q4) of the total 25‐OH‐D_3_ concentration.

In any situation, including the 3‐epi‐25‐OH‐D3 in total 25‐OH‐D concentration, attenuated the prevalence of vitamin D deficiency, insufficiency, and suboptimal levels in our results. Engelman et al. reported that only 1% of their population‐based sample of adults aged 21–74 years would have been classified as sufficient if the three epimers were included in the total 25‐OH‐D, but deficient (serum 25‐OH‐D < 50 nmol/L [20 ng/mL]) if it was excluded [32].

Chen et al. [17] reported that % 3‐epi‐25‐OH‐D_3_, not the level, may be a potential biomarker to reflect its pathological increase in multiple diseases. They noted that 15 specific diseases had a consistent association with % 3‐epi‐25‐OH‐D_3_, which meant that the % 3‐epi‐25‐OH‐D_3_ can serve as an indicator for increased generation of 3‐epi‐25‐OH‐D_3_ under pathological conditions with the pathological increase in 25‐OH‐D3 epimerization.

In our study, most blood markers, except for hs‐CRP, showed significant correlations with % 3‐epi‐25‐OH‐D_3_, after adjustment for age and gender. However, multiple linear regression analysis using these blood markers yielded very low *R* ^2^ values, as low as 0.06–0.07, indicating a weak statistical association (data not shown). Our results demonstrated that the % 3‐epi‐25‐OH‐D_3_ was inversely associated with Plt, BUN, creatinine, total cholesterol, and LDL cholesterol. These findings are consistent with a previous study in Korea [6], which reported an inverse association between total 25‐OH‐D and both total cholesterol and LDL cholesterol.

We did not include information on sun exposure, diet, medications, or supplements in this study; therefore, we are unable to assess the potential influence of these factors, which would be a limitation of our study.

Between 1988 and 2006, the NHANES produced nationally representative 25‐OH‐D data using RIA in the USA. Starting in 2007–2008, the CDC laboratory transitioned to a more accurate LC–MS/MS method, standardized to international reference materials, aligning with practices from other national surveys. Similarly, the UK Food Standards Agency recommended using LC–MS/MS for future National Diet and Nutrition Surveys. In Ireland, immunoassay data from the 2008–2010 Irish National Adult Nutrition Survey (NANS) were standardized to LC–MS/MS equivalents, with results compared to a complete reanalysis of survey samples using LC–MS/MS [31].

The serum mean 25‐OH‐D concentrations in the KNHANES 2010 were 19.58 ng/mL for males and 17.18 ng/mL for females. The overall mean concentrations in the KNHANES 2013 and 2014 were 16.44 and 16.30 ng/mL, respectively [33, 34]. All these measurements were obtained using the RIA method and were lower than the results from our KNHANES 2022–2023 study. The mean concentration of (25‐OH‐D_2_ + 25‐OH‐D_3_) in our research, excluding epimer concentrations, was 24.19 ± 11.23 ng/mL, also higher than those reported in previous studies.

Previous studies have shown discrepancies between LC–MS/MS and RIA (Diasorin, Stillwater, MN, USA) methods, yielding lower results than concentrations measured by LC–MS/MS [33]. LC–MS/MS demonstrates higher sensitivity and specificity than RIA, and DiaSorin RIA exhibits a proportional bias, measuring lower concentrations compared to LC–MS/MS as the actual concentration increases. However, in previous studies, the RIA method did not include the epimer form when measuring total 25‐OH‐D. On the other hand, the conventional LC–MS/MS method without epimer separation would have measured a “total 25‐OH‐D” with epimer form [16, 35].

We included SRM 972a (NIST, Gaithersburg, USA) in every batch of vitamin D measurement by LC–MS/MS as part of the internal QC program to ensure our accuracy. We also participated in the Vitamin D Standardization‐Certification Program (VDSCP) of the CDC to confirm the accuracy of our LC–MS/MS measurement in this national project. Our serum total 25‐OH‐D, 25‐OH‐D_3_, 25‐OH‐D_2_, and 3‐epi‐25‐OH‐D_3_ were reported to the CDC, where we received a very high “individual samples pass rate” and evaluation results compared to the DiaSorin method. This indicates that our LC‐MS/MS method for separating epimer forms is highly accurate, standardized, and traceable to the primary reference measurement procedure. It demonstrates that our method is more precise than DiaSorin RIA [36]. For quality control of all assays in the KNHANES project, including the vitamin D assay, “On‐Site Inspectors” oversaw the “Quality Control of the Clinical Laboratory for the Korea National Health and Nutrition Examination Survey (2022‐2024, 9th).” The inspector for the vitamin D assay has been overseeing rigorously quality control and quality management for the vitamin D assay in this project [37].

It is essential that the C3‐epimers can cause an overestimation of vitamin D status in routine laboratory tests. In the previous study in Korea, our LDT MS method separating 3‐epi‐25‐OH‐D_3_, Abbott, and Advia Centaur Siemens assay showed no cross‐reactivity and gave consistent total 25‐OH‐D results regardless of increasing 3‐epi‐25‐OH‐D_3_ concentrations. The MS methods, which do not separate 3‐epi‐25‐OH‐D_3_, and both the Beckman Coulter and Roche assays, all showed significant cross‐reactivity, with increasing degrees of interference correlating with increasing 3‐epi‐25‐OH‐D_3_ concentrations [26]. This is consistent with another study in Korea [25].

Epimer measurement remains challenging, making it difficult to incorporate into routine laboratory tests. The key question is whether epimers need to be measured in the future. Even though epimers may have limited clinical significance, they still influence the diagnosis of vitamin D deficiency, as demonstrated by the 4.1% difference between including and excluding epimers at 30 mg/mL in our study. Moving forward, especially in large‐scale national projects like the KNHANES, accurate measurement of vitamin D metabolites using mass spectrometry that separates epimer forms is essential. This approach, which aligns with global trends in national projects, will play a crucial role in conducting accurate public health assessments and guiding national policies for nutrition.

## Author Contributions

Sung‐Eun Cho, Jungsun Han, Rihwa Choi, Sang Gon Lee, and Hyung‐Doo Park conceived and designed the study. Jungsun Han investigated and measured vitamin D. Gayoung Chun conducted statistical analyses. Sung‐Eun Cho wrote the manuscript. Rihwa Choi and Sang Gon Lee provided feedback. Hyung‐Doo Park supervised the study. All authors have read and approved the final manuscript.

## Conflicts of Interest

The authors declare no conflicts of interest.

## Supporting information


**Data S1:** jcla70098‐sup‐0001‐Supinfo.docx.

**Data S2:** jcla70098‐sup‐0002‐Supinfo.docx.

## Data Availability

The data that support the findings of this study are available in the Supporting Information of this article.
