# Supplementary material for: Assessment of Serum 3‐Epi‐25‐Hydroxyvitamin D3 , 25‐Hydroxyvitamin D3 and 25‐Hydroxyvitamin D2 in the Korean Population With UPLC–MS/MS
Source: J Clin Lab Anal. 2025 Sep 12;39(19):e70098. doi: 10.1002/jcla.70098 (PMC12514969; doi:10.1002/jcla.70098)
Supplement: Supplementary file 1 — Data S1: jcla70098‐sup‐0001‐Supinfo.docx. [file JCLA-39-e70098-s002.docx]

Supplementary data Table S1. Chromatographic conditions of the UPLC

| Parameter | Condition |
| --- | --- |
| Column | Kinetex XB-C18 (2.1 × 150 mm, 2.6 μm, Phenomenex, Torrance, USA) |
| Column temperature | 30 °C |
| Mobile phase A | 0.025% additive in 50% methanol |
| Mobile phase B | 0.025% additive in 100% methanol |
| Gradient (isocratic) | 0-16 min: 45% A / 55% B |
| Washing solvent | 50% methanol (A), 100% methanol (B) |
| Flow rate | 0.15 mL/min |
| Injection volume | 2 μL |
| Autosampler temperature | 20 °C |
| Run time | 16 min |

Abbreviations: UPLC, ultra-performance liquid chromatography.

Supplementary data Table S2. MRM transitions and other setting conditions of the MS/MS

| Analyte | Q1  (m/z) | Q3  (m/z) | DP (V) | EP (V) | CE (V) | CXP (V) | CUR (psi) | CAD (psi) | Temp (°C) | Dwell Time (msec) |
| --- | --- | --- | --- | --- | --- | --- | --- | --- | --- | --- |
| 25-OH-D_2_ | 619.4 Da | 298.1 Da | 80 | 10 | 27 | 13 | 30 | 9 | 600 | 150 |
| 25-OH-D2 IS  (^2^H_3_-25-OH-D_2_) | 622.4 Da | 301.1 Da | 80 | 10 | 27 | 13 | 30 | 9 | 600 | 150 |
| 25-OH-D_3_ | 607.4 Da | 298.1 Da | 80 | 10 | 29 | 15 | 30 | 9 | 600 | 150 |
| 25-OH-D_3_ IS  (^2^H_3_-25-OH-D_3_) | 610.4 Da | 301.1 Da | 80 | 10 | 29 | 15 | 30 | 9 | 600 | 150 |
| 3-epi-25-OH-D_3_ | 607.4 Da | 298.1 Da | 80 | 10 | 29 | 15 | 30 | 9 | 600 | 150 |
| 3-epi-25-OH-D_3_ IS  (^2^H_3_-3-epi-25-OH-D_3_) | 610.4 Da | 301.1 Da | 80 | 10 | 29 | 15 | 30 | 9 | 600 | 150 |

Abbreviations: MRM, multiple reaction monitoring; IS, internal standard; DP, de-clustering potential; EP, exit potential; CE, collision energy; CXP, collision cell exit potential; CUR, curtain gas; CAD, collision gas; Temp, temperature.

Supplementary data Table S3. Intra- and inter-run precision and accuracy

| Analyte | SRM  972a  Level | Target values (ng/mL) | Precision | | | | Accuracy | |
| --- | --- | --- | --- | --- | --- | --- | --- | --- |
|  |  |  | Intra-run (N=5) | | Inter-run (N=20) | |  |  |
|  |  |  | Mean  concentrations (ng/mL) | % CV | Mean concentrations (ng/mL) | % CV | Mean concentrations (ng/mL) | % Bias |
| 25-OH-D_2_ | Level 2 | 0.81 | 0.88 | 1.14 | 0.84 | 2.38 | 0.85 | 4.94 |
|  | Level 3 | 13.2 | 13.91 | 0.58 | 14.11 | 1.42 | 14.09 | 6.74 |
| 25-OH-D_3_ | Level 1 | 28.8 | 29.12 | 1.85 | 29.24 | 2.05 | 28.63 | -0.59 |
|  | Level 2 | 18.1 | 18.35 | 3.05 | 18.41 | 2.61 | 18.07 | -0.17 |
|  | Level 3 | 19.8 | 19.98 | 2.70 | 19.92 | 2.26 | 19.52 | -1.41 |
|  | Level 4 | 29.4 | 30.25 | 3.14 | 29.90 | 3.01 | 29.70 | 1.02 |
| 3-epi-25-OH-D_3_ | Level 1 | 1.81 | 1.66 | 1.81 | 1.84 | 4.89 | 1.75 | -3.31 |
|  | Level 2 | 1.28 | 1.17 | 1.71 | 1.25 | 4.00 | 1.22 | -4.69 |
|  | Level 4 | 26.0 | 23.41 | 1.67 | 26.65 | 3.60 | 26.51 | 1.96 |

Supplementary data Table S4-1. Monthly quality control chart summary of 25-OH-D_2_ using

SRM 972a in 2024

| Month | No. of batches | SRM 972a  Level | Target values (ng/mL) | Mean concentrations (ng/mL) | % Bias | % CV | % Bias  measured range |
| --- | --- | --- | --- | --- | --- | --- | --- |
| January | 1 | Level 2 | 0.81 | 0.88 | 8.64 | - | - |
|  |  | Level 3 | 13.2 | 14.98 | 13.48 | - | - |
| February | 12 | Level 2 | 0.81 | 0.86 | 6.17 | 4.65 | -1.23~14.81 |
|  |  | Level 3 | 13.2 | 14.94 | 13.18 | 1.54 | 8.94~14.92 |
| March | 12 | Level 2 | 0.81 | 0.86 | 6.17 | 4.65 | 0.00~12.35 |
|  |  | Level 3 | 13.2 | 14.80 | 12.12 | 2.16 | 8.79~14.85 |
| April | 9 | Level 2 | 0.81 | 0.85 | 4.94 | 3.53 | 0.00~9.88 |
|  |  | Level 3 | 13.2 | 15.05 | 14.02 | 0.53 | 13.33~15.00 |
| May | 9 | Level 2 | 0.81 | 0.87 | 7.41 | 3.45 | 1.23~11.11 |
|  |  | Level 3 | 13.2 | 15.06 | 14.09 | 0.73 | 12.12~15.00 |
| June | 7 | Level 2 | 0.81 | 0.88 | 8.64 | 2.27 | 6.17~12.35 |
|  |  | Level 3 | 13.2 | 14.88 | 12.73 | 1.34 | 10.68~14.24 |
| July | 9 | Level 2 | 0.81 | 0.89 | 9.88 | 3.37 | 6.17~14.81 |
|  |  | Level 3 | 13.2 | 14.61 | 10.68 | 2.33 | 6.21~14.02 |
| August | 6 | Level 2 | 0.81 | 0.90 | 11.11 | 2.22 | 7.41~13.58 |
|  |  | Level 3 | 13.2 | 14.95 | 13.26 | 0.67 | 12.42~14.09 |
| September | 7 | Level 2 | 0.81 | 0.88 | 8.64 | 3.41 | 4.94~13.58 |
|  |  | Level 3 | 13.2 | 15.04 | 13.94 | 0.80 | 12.80~15.00 |
| October | 10 | Level 2 | 0.81 | 0.88 | 8.64 | 2.27 | 4.94~12.35 |
|  |  | Level 3 | 13.2 | 14.55 | 10.23 | 1.86 | 7.65~13.64 |
| November | 9 | Level 2 | 0.81 | 0.86 | 6.17 | 4.65 | -4.94~12.35 |
|  |  | Level 3 | 13.2 | 14.65 | 10.98 | 1.77 | 7.50~14.24 |
| December | 7 | Level 2 | 0.81 | 0.86 | 6.17 | 3.49 | 1.23~11.11 |
|  |  | Level 3 | 13.2 | 14.36 | 8.79 | 4.81 | -0.91~14.39 |

Supplementary data Table S4-2. Monthly quality control chart summary of 25-OH-D_3_ using

SRM 972a in 2024

| Month | No. of batches | SRM 972a  Level | Target values (ng/mL) | Mean concentrations (ng/mL) | % Bias | % CV | % Bias  measured  range |
| --- | --- | --- | --- | --- | --- | --- | --- |
| January | 1 | Level 1 | 28.8 | 30.46 | 5.76 | - | - |
|  |  | Level 2 | 18.1 | 19.17 | 5.91 | - | - |
|  |  | Level 3 | 19.8 | 20.65 | 4.29 | - | - |
|  |  | Level 4 | 29.4 | 30.85 | 4.93 | - | - |
| February | 12 | Level 1 | 28.8 | 30.79 | 6.91 | 3.70 | -0.80~11.53 |
|  |  | Level 2 | 18.1 | 19.20 | 6.08 | 2.45 | 2.04~9.28 |
|  |  | Level 3 | 19.8 | 21.19 | 7.02 | 2.60 | 1.36~10.56 |
|  |  | Level 4 | 29.4 | 31.33 | 6.56 | 2.84 | -0.07~10.44 |
| March | 12 | Level 1 | 28.8 | 30.27 | 5.10 | 2.38 | 1.94~9.44 |
|  |  | Level 2 | 18.1 | 18.90 | 4.42 | 2.86 | 0.33~9.89 |
|  |  | Level 3 | 19.8 | 20.65 | 4.29 | 2.32 | 0.45~7.73 |
|  |  | Level 4 | 29.4 | 30.87 | 5.00 | 2.46 | 1.80~9.69 |
| April | 9 | Level 1 | 28.8 | 31.02 | 7.71 | 1.97 | 3.16~10.42 |
|  |  | Level 2 | 18.1 | 19.38 | 7.07 | 1.29 | 4.42~9.28 |
|  |  | Level 3 | 19.8 | 21.17 | 6.92 | 1.70 | 2.68~9.09 |
|  |  | Level 4 | 29.4 | 31.48 | 7.07 | 1.87 | 2.72~9.35 |
| May | 9 | Level 1 | 28.8 | 30.31 | 5.24 | 3.17 | -2.43~9.83 |
|  |  | Level 2 | 18.1 | 19.08 | 5.41 | 2.78 | 1.10~9.28 |
|  |  | Level 3 | 19.8 | 20.87 | 5.40 | 2.30 | 0.86~9.09 |
|  |  | Level 4 | 29.4 | 31.13 | 5.88 | 2.83 | 2.69~10.65 |
| June | 7 | Level 1 | 28.8 | 30.41 | 5.59 | 0.92 | 4.31~7.01 |
|  |  | Level 2 | 18.1 | 18.82 | 3.98 | 2.23 | 0.33~6.63 |
|  |  | Level 3 | 19.8 | 20.65 | 4.29 | 1.69 | 2.02~6.31 |
|  |  | Level 4 | 29.4 | 30.91 | 5.14 | 1.68 | 1.60~6.80 |
| July | 9 | Level 1 | 28.8 | 30.37 | 5.45 | 2.96 | -0.62~8.72 |
|  |  | Level 2 | 18.1 | 19.13 | 5.69 | 1.88 | 2.60~8.62 |
|  |  | Level 3 | 19.8 | 20.85 | 5.30 | 2.54 | -0.15~8.48 |
|  |  | Level 4 | 29.4 | 31.12 | 5.85 | 1.80 | 3.10~8.57 |
| August | 6 | Level 1 | 28.8 | 30.12 | 4.58 | 2.12 | 1.22~6.53 |
|  |  | Level 2 | 18.1 | 18.80 | 3.87 | 1.76 | 1.38~6.41 |
|  |  | Level 3 | 19.8 | 20.90 | 5.56 | 2.39 | 2.78~8.69 |
|  |  | Level 4 | 29.4 | 30.91 | 5.14 | 2.04 | 2.41~6.94 |
| September | 7 | Level 1 | 28.8 | 29.99 | 4.13 | 4.20 | -1.53~9.44 |
|  |  | Level 2 | 18.1 | 19.44 | 7.40 | 2.16 | 2.60~9.78 |
|  |  | Level 3 | 19.8 | 20.85 | 5.30 | 3.50 | 0.91~10.35 |
|  |  | Level 4 | 29.4 | 31.08 | 5.71 | 3.25 | -1.22~9.18 |
| October | 10 | Level 1 | 28.8 | 29.78 | 3.40 | 2.92 | -0.62~9.17 |
|  |  | Level 2 | 18.1 | 18.53 | 2.38 | 3.83 | -2.60~7.18 |
|  |  | Level 3 | 19.8 | 20.45 | 3.28 | 2.98 | -0.71~9.44 |
|  |  | Level 4 | 29.4 | 30.36 | 3.27 | 2.67 | -0.31~6.80 |
| November | 9 | Level 1 | 28.8 | 29.60 | 2.78 | 3.14 | -2.95~6.01 |
|  |  | Level 2 | 18.1 | 18.66 | 3.09 | 3.59 | -3.87~7.68 |
|  |  | Level 3 | 19.8 | 20.38 | 2.93 | 2.26 | -0.20~6.26 |
|  |  | Level 4 | 29.4 | 30.18 | 2.65 | 2.95 | -3.37~6.77 |
| December | 7 | Level 1 | 28.8 | 30.53 | 6.01 | 1.80 | 2.40~8.09 |
|  |  | Level 2 | 18.1 | 18.63 | 2.93 | 4.13 | -4.14~8.78 |
|  |  | Level 3 | 19.8 | 20.05 | 1.26 | 4.59 | -6.36~9.34 |
|  |  | Level 4 | 29.4 | 30.01 | 2.07 | 3.23 | -3.13~6.33 |

Supplementary data Table S4-3. Monthly quality control chart summary of 3-epi-25-OH-D_3_

using SRM 972a in 2024

| Month | No. of batches | SRM 972a  Level | Target values (ng/mL) | Mean concentrations (ng/mL) | % Bias | % CV | % Bias  measured  range |
| --- | --- | --- | --- | --- | --- | --- | --- |
| January | 1 | Level 1 | 1.81 | 1.86 | 2.76 | - | - |
|  |  | Level 2 | 1.28 | 1.31 | 2.34 | - | - |
|  |  | Level 4 | 26.0 | 25.55 | -1.73 | - | - |
| February | 12 | Level 1 | 1.81 | 1.81 | 0.00 | 4.97 | -7.18~9.94 |
|  |  | Level 2 | 1.28 | 1.23 | -3.91 | 5.69 | -12.50~3.91 |
|  |  | Level 4 | 26.0 | 25.29 | -2.73 | 6.09 | -12.50~7.62 |
| March | 12 | Level 1 | 1.81 | 1.80 | -0.55 | 4.44 | -6.08~9.39 |
|  |  | Level 2 | 1.28 | 1.24 | -3.13 | 5.65 | -12.50~9.37 |
|  |  | Level 4 | 26.0 | 26.49 | 1.88 | 3.21 | -4.85~6.00 |
| April | 9 | Level 1 | 1.81 | 1.80 | -0.55 | 5.00 | -9.39~7.18 |
|  |  | Level 2 | 1.28 | 1.21 | -5.47 | 4.13 | -13.28~0.78 |
|  |  | Level 4 | 26.0 | 26.43 | 1.65 | 4.46 | -3.62~9.35 |
| May | 9 | Level 1 | 1.81 | 1.87 | 3.31 | 6.42 | -7.18~14.92 |
|  |  | Level 2 | 1.28 | 1.28 | 0.00 | 8.59 | -8.59~13.28 |
|  |  | Level 4 | 26.0 | 27.16 | 4.46 | 5.93 | -3.73~14.96 |
| June | 7 | Level 1 | 1.81 | 1.86 | 2.76 | 4.84 | -6.08~9.94 |
|  |  | Level 2 | 1.28 | 1.30 | 1.56 | 4.62 | -4.69~7.81 |
|  |  | Level 4 | 26.0 | 26.84 | 3.23 | 5.33 | -4.96~9.96 |
| July | 9 | Level 1 | 1.81 | 1.86 | 2.76 | 3.23 | -4.42~6.08 |
|  |  | Level 2 | 1.28 | 1.30 | 1.56 | 5.38 | -7.03~12.50 |
|  |  | Level 4 | 26.0 | 25.81 | -0.73 | 2.87 | -4.23~3.08 |
| August | 6 | Level 1 | 1.81 | 1.79 | -1.10 | 6.15 | -9.94~7.18 |
|  |  | Level 2 | 1.28 | 1.24 | -3.13 | 5.65 | -9.38~3.91 |
|  |  | Level 4 | 26.0 | 24.61 | -5.35 | 7.07 | -14.08~3.08 |
| September | 7 | Level 1 | 1.81 | 1.90 | 4.97 | 3.68 | -3.31~7.73 |
|  |  | Level 2 | 1.28 | 1.31 | 2.34 | 6.11 | -8.59~9.37 |
|  |  | Level 4 | 26.0 | 28.10 | 8.08 | 3.81 | 1.42~12.96 |
| October | 10 | Level 1 | 1.81 | 1.86 | 2.76 | 6.45 | -6.63~14.92 |
|  |  | Level 2 | 1.28 | 1.28 | 0.00 | 7.81 | -9.38~13.28 |
|  |  | Level 4 | 26.0 | 26.82 | 3.15 | 6.56 | -9.88~13.31 |
| November | 9 | Level 1 | 1.81 | 1.81 | 0.00 | 7.18 | -12.71~7.18 |
|  |  | Level 2 | 1.28 | 1.27 | -0.78 | 5.51 | -6.25~9.37 |
|  |  | Level 4 | 26.0 | 25.73 | -1.04 | 4.31 | -6.31~7.31 |
| December | 7 | Level 1 | 1.81 | 1.88 | 3.87 | 2.66 | -0.55~7.18 |
|  |  | Level 2 | 1.28 | 1.26 | -1.56 | 5.56 | -9.38~6.25 |
|  |  | Level 4 | 26.0 | 25.75 | -0.96 | 4.50 | -7.46~4.19 |


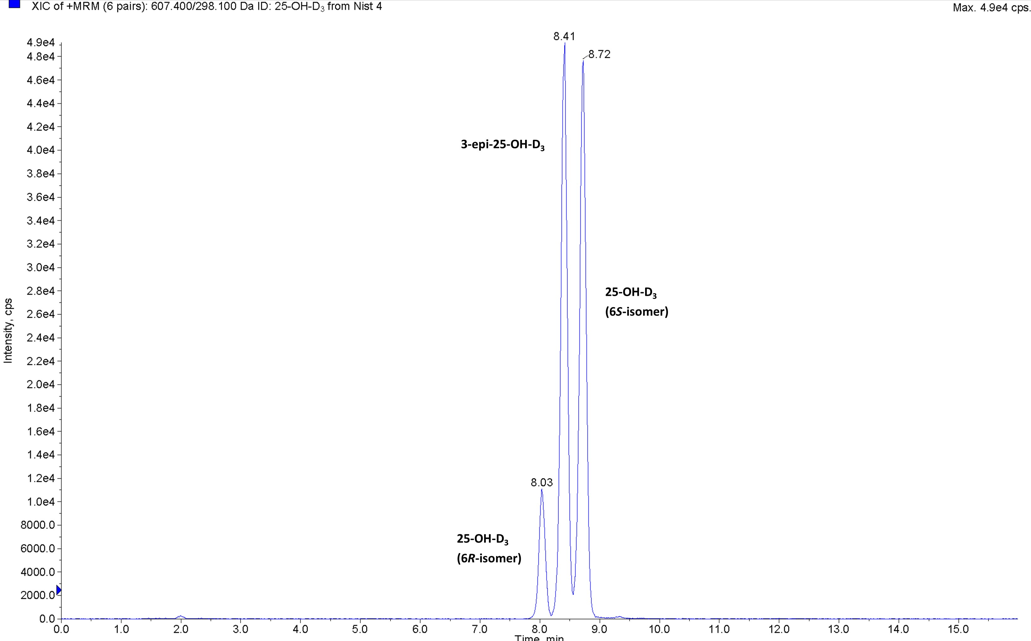


Supplementary data Fig. S1. Tandem mass spectrometry chromatogram showing the separation of the 6S- and 6R-isomers of 25-OH-D3 and 3-epi-OH-D_3_ in NIST SRM 972a Level 4.
